# Supplementary figures and images for: Legume NCRs and nodule-specific defensins of actinorhizal plants—Do they share a common origin?
Source: PLoS One. 2022 Aug 18;17(8):e0268683. doi: 10.1371/journal.pone.0268683 (PMC9387825; doi:10.1371/journal.pone.0268683)

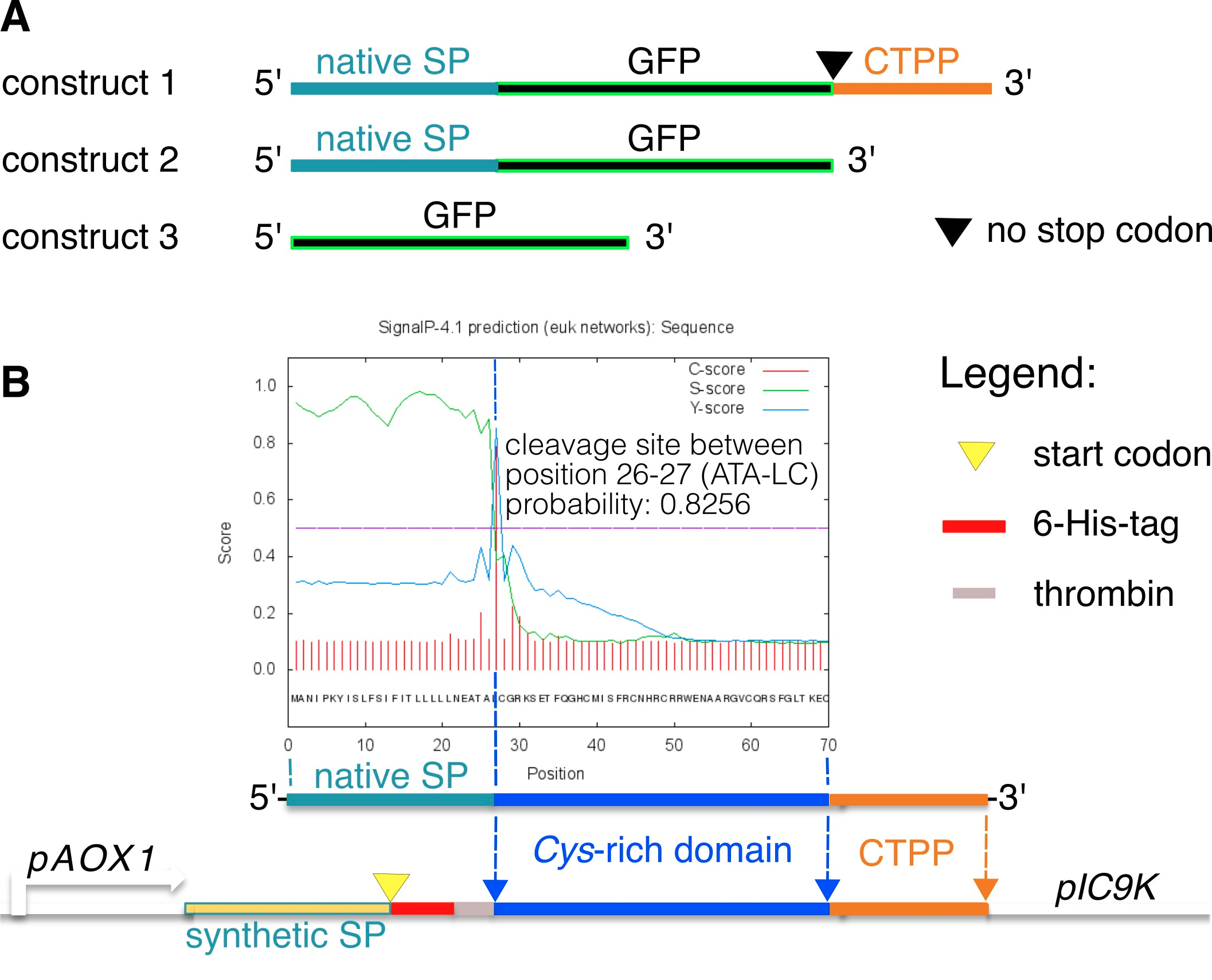

Supplement: S1 Fig — Schematic presentation of constructs used for subcellular localization (A) and heterologous production (B) of DgDef1ΔSP. Panel A displays the three GFP chimeras generated for Agrobacterium tumefaciens-mediated transformation of Nicotiana benthamiana (see Materials and Methods). Panel B illustrates the strategy employed to prepare a synthetic cassette for expression of DgDef1ΔSP in Pichia pastoris. SignalP (v.4.1) predicted a signal peptide (SP) in the DgDef1 ORF (see plot). Taking advantage of pIC9K as a secretion vector, the native DgDef1 SP was replaced by the synthetic SP located downstream of the strong promoter AOX1. A 6-His-tag and a thrombin cleavage site were engineered (see Materials and Methods). Note: Depicted domains are not on scale. (TIFF) [file pone.0268683.s001.tiff]

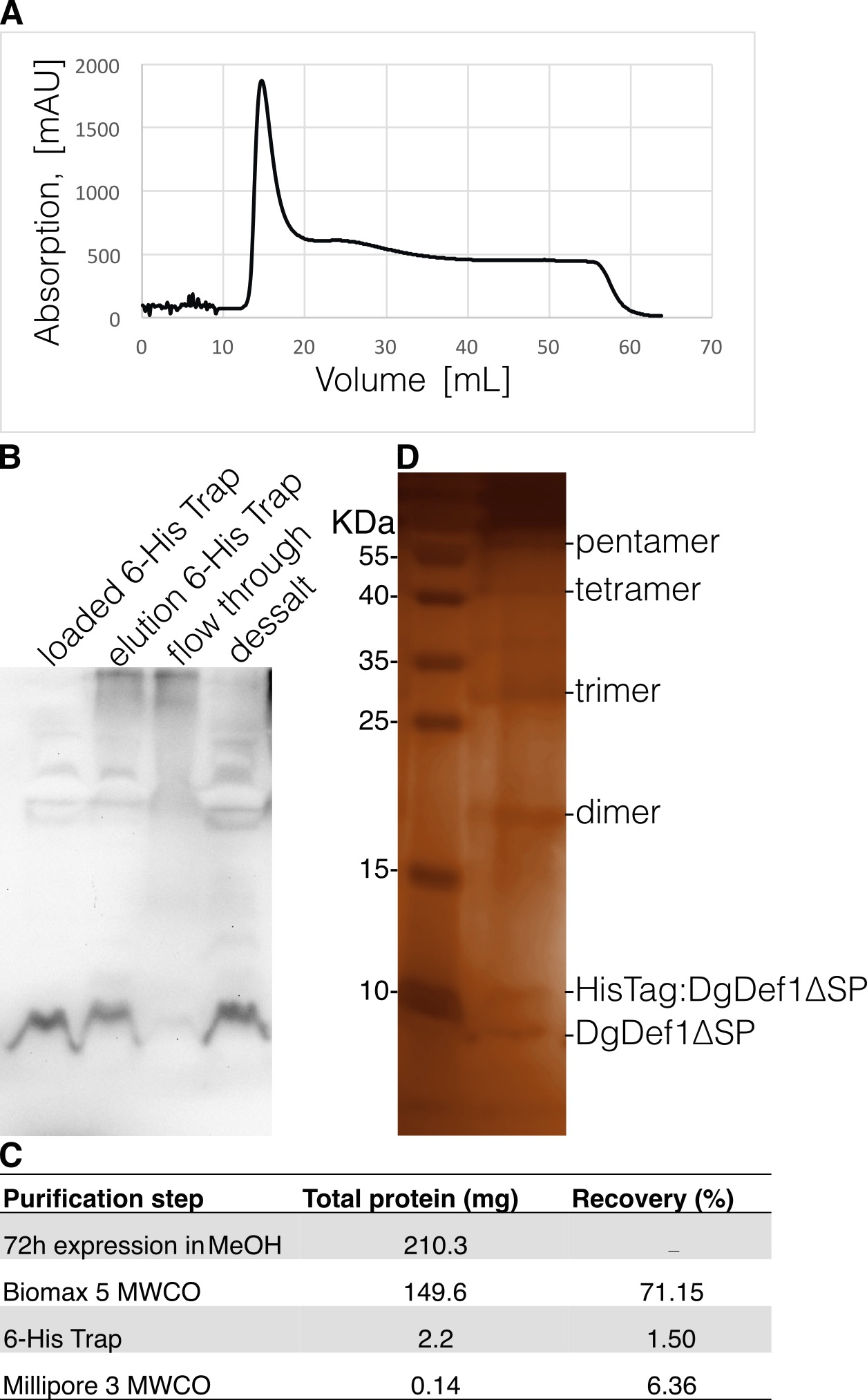

Supplement: S4 Fig — (A) Chromatogram showing the elution peak of DgDef1ΔSP. (B) Immunoblotting analysis summarizing the different purification steps. (C) Purification table. (D) Silver staining showing oligomeric structures of purified DgDef1ΔSP. (TIFF) [file pone.0268683.s004.tiff]

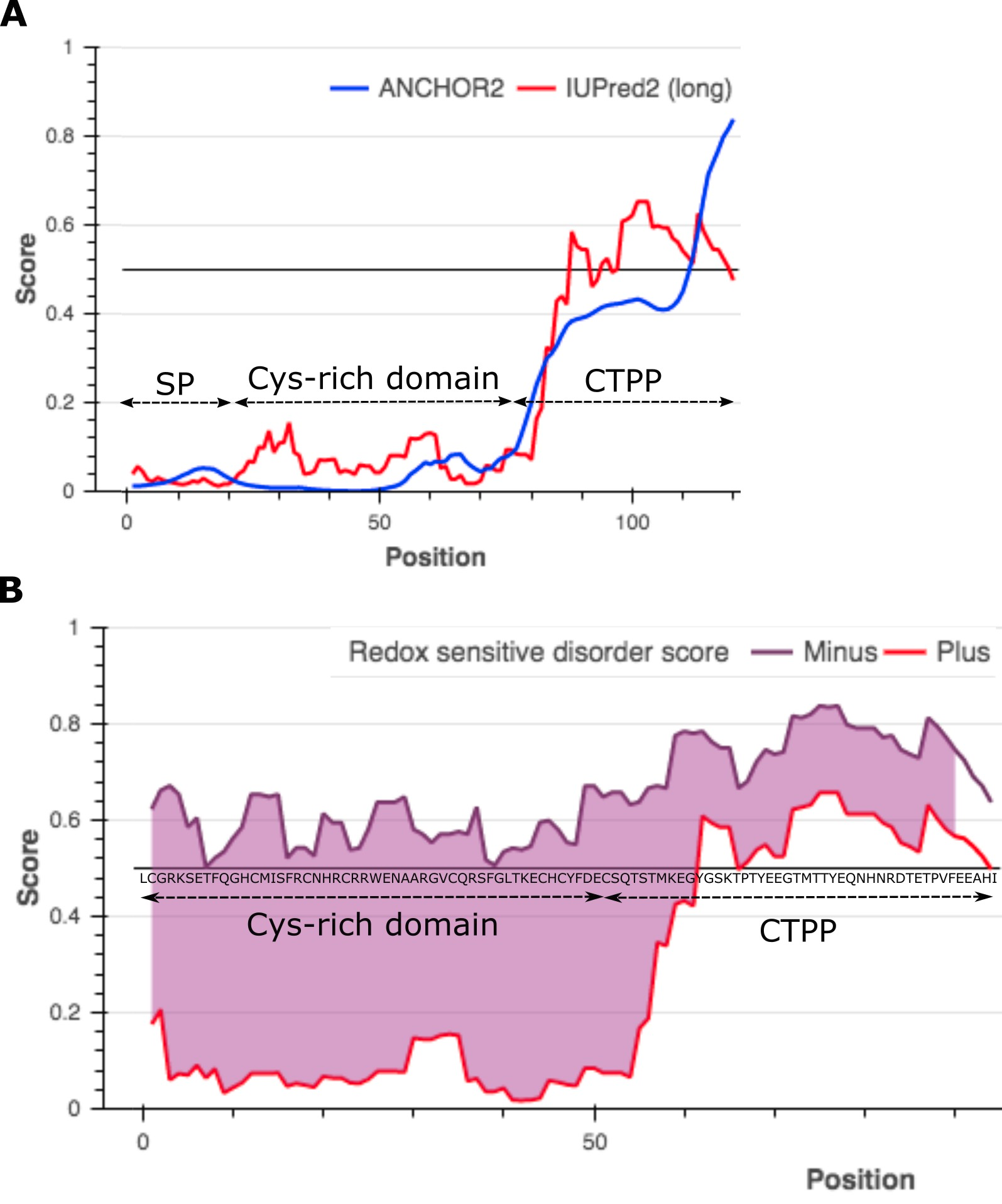

Supplement: S6 Fig — (A) The predicted output of IUPred2 (red) and ANCHOR2 (blue) for DgDef1. (B) Redox-state-dependent IUPred2 prediction for the Pichia-produced peptide DgDef1ΔSP. The estimated sensitivity of the disorder tendency is marked in purple. (TIFF) [file pone.0268683.s006.tiff]
